# ‘Antibiotic footprint’ as a communication tool to aid reduction of antibiotic consumption—authors’ response

**DOI:** 10.1093/jac/dkz372

**Published:** 2019-09-03

**Authors:** Direk Limmathurotsakul, Jonathan A T Sandoe, David C Barrett, Michael Corley, Li Yang Hsu, Marc Mendelson, Peter Collignon, Ramanan Laxminarayan, Sharon J Peacock, Philip Howard

**Affiliations:** 1 Mahidol-Oxford Tropical Medicine Research Unit, Faculty of Tropical Medicine, Mahidol University, Bangkok, Thailand; 2 Department of Tropical Hygiene, Faculty of Tropical Medicine, Mahidol University, Bangkok, Thailand; 3 Centre for Tropical Medicine and Global Health, University of Oxford, Oxford, UK; 4 University of Leeds/Leeds Teaching Hospitals NHS Trust, Leeds, UK; 5 British Society of Antimicrobial Chemotherapy, Birmingham, UK; 6 Bristol Veterinary School, University of Bristol, Bristol, UK; 7 Saw Swee Hock School of Public Health, National University of Singapore and National University Health System, 12 Science Drive 2, Singapore; 8 National Centre for Infectious Diseases, Moulmein Road, Singapore; 9 Division of Infectious Diseases & HIV Medicine, Department of Medicine, University of Cape Town, Cape Town, South Africa; 10 International Society for Infectious Diseases, Brookline, MA, USA; 11 Infectious Diseases and Microbiology, Canberra Hospital, Canberra, Australia; 12 Medical School, Australian National University, Acton, Australia; 13 Center for Disease Dynamics, Economics & Policy, New Delhi, 110024, India; 14 Princeton Environmental Institute, Princeton, NJ, USA; 15 Department of Medicine, University of Cambridge, Cambridge, UK

Sir,

We thank Lipsitch and Shaman^1^ for their comments on our article^2^ on the antibiotic footprint, which we proposed as a simple means of communication to aid public understanding. The total amount of consumption of different antibiotics could predominantly affect the development and spread of antibiotic resistance in the sector where the antibiotics are used. However, there are spillovers of antibiotic resistance into other sectors,^3^ which is why we believe the concept of a total footprint is useful. If we reduce antibiotic use in the agriculture sector it may result in less resistance in bacteria colonizing and/or infecting people.^3^ We agree that the antibiotic footprint could be improved by better taking account of the impact of antibiotic consumption in different sectors and for different types of antibiotics, and would wish to see research in this area in the future.^2^ On the issue of how antibiotic volumes should best be expressed, as data and epidemiological and modelling studies become increasingly available, researchers may be able to convert tonnes of different types of antibiotics used in different sectors to penicillin equivalent (just for a notion and an example). The complex models for converting each specific drug for each specific sector could be widely debated and later decided upon among researchers and stakeholders in order to support communication with the public.

We proposed that antimicrobial consumption should be reduced to a minimum, not to zero, and we agree that access to antibiotics for those who have to use them must not be compromised.^2^ The antibiotic footprint could also be used, however, to promote this concept and stimulate people to ask whether underuse or poor access to antibiotics occurs in some areas. For example, on average in 2015, a person living in the Netherlands, where misuse of antibiotics in humans and problems of access to antibiotics among those who have to use them are very low, directly consumed about 3.3 g of antibiotic.^2^^,^^4^ A person living in Burundi, Azerbaijan or the Philippines directly consumed ∼1.6, 2.8 or 3.0 g of antibiotics, respectively, in 2015,^2^^,^^4^ and underuse or poor access of antibiotics may be a major problem requiring urgent action. Overuse of antibiotics might also occur in some areas, or official data on antibiotic consumption might be incomplete. A tool such as the antibiotic footprint could be used to communicate with the public and stakeholders to promote studies and actions.

We are aware of the potential concerns that clinicians, veterinarians or farmers could switch antibiotic type to minimize the total kilograms/tonnes used; however, many actions could be taken to monitor and discourage inappropriate changes. First, data on each antibiotic’s usage in each sector from each country should be made openly available. Additional advanced metrics (such as DDD, mg/population correction unit, mg/kg, daily dose metrics and course dose metrics) can then be used by researchers and stakeholders to evaluate the changes in usage in greater detail. Second, actions from governments and stakeholders are always needed. For example, the USA has reported a 43% decrease in antibiotic consumption in animals from 9702 tonnes in 2015 to 5559 tonnes in 2017.^5^^,^^6^ There was also a decrease in most of the medically important antibiotics as the FDA’s new rules require veterinary oversight of usage, and these drugs can no longer be purchased over the counter.^5^^,^^6^ This could be used to inform stakeholders in many low- and middle-income countries that reducing antibiotic consumption in animals without an increase in animal health or food safety problems is possible.^6^ Nonetheless, a 24% increase in fluoroquinolone use from 18.5 tonnes in 2016 to 22.9 tonnes in 2017 was also observed,^5^ and additional actions from the government and stakeholders are being taken to further reduce antibiotic consumption in animals to a minimum.^6^ Another example of a concept used by a government is the *UK One Health Report 2013–2017*, which highlights a reduction of 19% in the total tonnage used by humans and in animals, with animal use contributing a 35% reduction and humans a 6% reduction (Figure 1).^7^ The report also presents data showing that the proportion used in humans rose to 64% from 55% during the period covered by the report. These data assisted in the development of goals within the *UK Antimicrobial Resistance National Action Plan 2019–2024*.^8^

**Figure 1:**
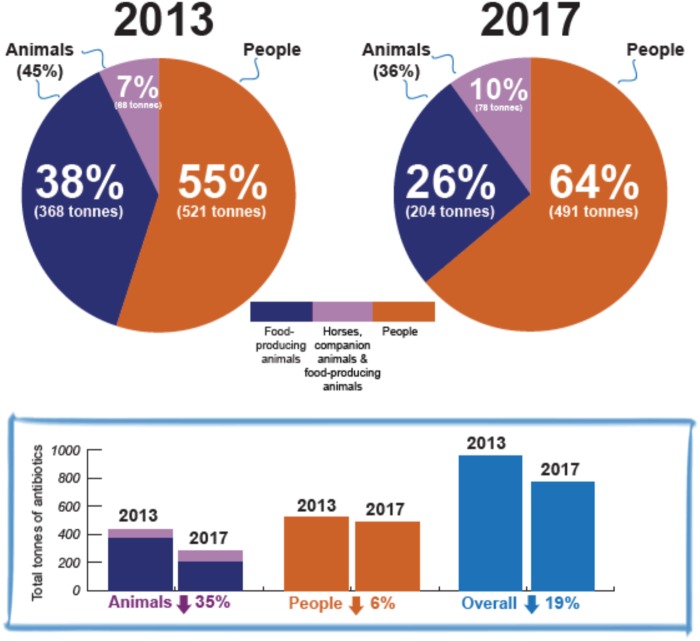
Antibiotic consumption in humans and animals. Reproduced from https://www.gov.uk/^7^ under the terms of the Open Government License v3.0. This figure appears in colour in the online version of *JAC* and in black and white in the print version of *JAC*.

## Transparency declarations

None to declare.
